# Nano-Emulsification Potentiates Tea Tree Oil Bioactivity: High-Stability Formulation for Dual Antimicrobial and Antioxidant Food Preservation

**DOI:** 10.3390/foods14193405

**Published:** 2025-10-01

**Authors:** Congnan Cen, Xinxuan Wang, Huan Li, Song Miao, Jian Chen, Yanbo Wang

**Affiliations:** 1 Key Laboratory of Microbiological Metrology, Measurement & Bio-Product Quality Security, State Administration for Market Regulation, College of Life Science, China Jiliang University, Hangzhou 310018, China; 24a0904183@cjlu.edu.cn; 2 Food Safety Key Laboratory of Zhejiang Province, School of Food Science and Biotechnology, Zhejiang Gongshang University, Hangzhou 310018, China; wang2385876494@outlook.com (X.W.); huanli@zjsu.edu.cn (H.L.); 3 School of Food and Health, Beijing Technology and Business University, Beijing 100048, China; song.miao@teagasc.ie; 4 Teagasc Food Research Centre, Moorepark, P61 C996 Cork, Ireland

**Keywords:** nanoemulsions, antibacterial property, tea tree essential oil, TEM

## Abstract

Essential oils play important roles in the modern food industry as additives and spices. At the same time, most essential oils have broad-spectrum bacteriostatic properties and can be used as natural antimicrobial materials. However, the application of essential oils is limited due to their strong volatility and insolubility in aqueous substrates. In this study, we used ultrasonic emulsification, carboxymethyl chitosan, and Tween 80 to formulate tea tree essential oil (TTO) nanoemulsions with high stability. With a minimum diameter of about 51 nm (PDI = 0.236 ± 0.021) post-emulsification, the TTO nanoemulsions disperse effectively in the drainage system and exhibit good stability after 14 days of storage. In addition, the bioactivity (antibacterial and antioxidant) of TTO nanoemulsions was significantly enhanced following emulsification, as evidenced by MIC and DPPH assays, indicating that nano-emulsification is beneficial to the development of various essential oils. TTO nanoemulsions can be used as a new food preservative to control the growth of bacteria and prevent the deterioration of food via oxidation.

## 1. Introduction

Microbial proliferation in food can trigger spoilage and toxic amine production through metabolic activities, which may result in a decrease in economic and safety factors [1]. In production practices, it has been found that physical sterilization methods may lead to the loss of nutrients in food. Chemical antimicrobial agents have also been found to be potentially harmful to human health in recent years of research [2]. In this situation, the use of natural substances as additives seems to be more relevant to the needs of production and consumers [3,4]. Plant essential oils, which possess strong natural antibacterial and antioxidant activity, appear to be good-quality natural alternative additives [5,6].

Chemical preservatives generally consist of a single component molecule. In contrast, essential oils are a blend of natural aromatic substances extracted from plant material by distillation or other extraction methods, and generally contain alcohols, aldehydes, acids, phenols, acetones, terpenes, etc. [7]. In fact, essential oils have been used for a long time in pharmaceuticals, cosmetics, and fragrances, with great success [8,9]. Essential oils are also recognized by the FDA as having regulatory safety status (GRAS) and can be used as natural food preservatives as a means of extending the shelf life of foods [10]. Some essential oils are already present as additives in some baked goods and pre-packaged foods [11]. Because of the broad spectrum of antimicrobial activity of plant oils, there has been an increase in research into their use as an alternative to chemical antibacterial agents in recent years [12,13].

However, there are some equally vexing physical properties of essential oils. Their low water solubility can make it difficult to maintain good physical stability in water-based foods, due to their aromatic compound content [14]. Their high volatility and strong odor may lead to sensory degradation of the food product, which certainly limits the scope of its application in the food industry [15,16]. In addition, although essential oils and their components show significant antibacterial activity, the number of essential oils that would need to be added to achieve the same effect would be on the high side due to the complex composition of food components [17,18]. Based on this, some studies have also pointed out that high concentrations of essential oils may have toxic effects on humans [19]. Therefore, new combinations of methods and strategies are needed to improve the physical properties and biological activity of essential oils when used as food preservatives. The use of nano-encapsulation, including nano-emulsification, micro-encapsulation, hydrogels, etc., seems to be a very high-quality way to handle substances that are difficult to dissolve with low bioavailability. Nanoemulsions are efficient hydrophobic delivery systems that disperse non-soluble substances in solvent dispersions [20,21], thus reducing the frequency of degradation, oxidation, or interactions with other food ingredients. By increasing the dispersibility of nanoemulsions, bioactive ingredients can be easily incorporated into food matrices after being encapsulated [22]. Nanoemulsions appear to be a promising alternative formulation for essential oils in this respect [23].

The oil of the tea tree plant is a natural hydrophobic antioxidant obtained from its seeds and leaves [24]. The most important chemical constituents are terpinene, pinene, limonene, cineole, terpineol, and cumene. There are several compounds in TTO, including polyphenols, which are toxic to certain bacteria, viruses, and fungi [25,26]. It mainly inhibits bacterial growth by scavenging reactive oxygen species and damaging the cell membrane [17]. Thus, TTO is widely used in biomedical fields for reducing inflammation, lowering oxidative stress, and improving wound healing [27]. However, TTOs suffer from the common problems of plant essential oils, such as poor stability, low bioavailability, and high volatility [28]. We can then use nano-encapsulation methods to improve its stability, bioavailability and bioefficacy. Although there have been some studies on nanoemulsions related to TTO in recent years, due to their strong volatility, the stability of their storage also needs to be paid attention to [29,30]. Polysaccharides, as a better biological matrix, can be used as a better emulsifying material [31,32]. Carboxymethyl chitosan is widely used in food and drug delivery with its excellent biocompatibility [33]. Due to its amphiphilicity and double charge, it can be used as an excellent carrier to help improve the emulsification of essential oils and their biological activity [34]. In this study, we aimed to improve the physicochemical properties of TTO essential oil through emulsification and to expand its potential applications.

## 2. Materials and Methods

### 2.1. Materials

Tea tree oil (TTO) was selected as the core component. TTO and lecithin were purchased from Aladdin (Shanghai, China). Carboxymethyl chitosan (CMCS) was purchased from West Asia Co., Ltd. (Shandong, China). Other chemicals were purchased from Macklin (Shanghai, China). This experiment used deionized water purified by Mili-Q (Millipore Co. in Bedford, MA, USA).

### 2.2. TTO Nanoemulsions Preparation

A mixture of TTO and 1% CMCS (in deionized water) at ratios of 1:1 (*v*/*v*) were used as the core materials. Polysorbate 80 (Tween 80) at 2%, 4%, and 6% (*v*/*v*) were then added as the surfactant. Lecithin of 0.5%, 1%, and 1.5% (*v*/*v*) were added as the cosurfactant. The deionized water was added to achieve a final volume of 60 mL. The mixture was stirred with 600 r/min for 45 min. The mixture was pre-mixed with a high-speed homogenizer at 10,000 rpm for 6 min at 25 °C. These coarse emulsions were finely dispersed with ultrasonic emulsification at a power of 420 W for 16, 24, and 32 min (work time: rest time = 1:1).

### 2.3. Droplet Size Measurement

The droplet size and polymer dispersity index of TTO nanoemulsions were performed using a Malvern Zetasizer (Malvern, UK) (Refractive index 1.3339, transmittance 0.01) [35].

### 2.4. Transmission Electron Microscope (TEM)

A transmission electron microscope (FEI Tecnai 12, FEI Company, Hillsboro, OR, USA) was used to observe the size and distribution status of the nanoemulsions’ droplets. Images of the nanoemulsions were taken at 25,000×~30,000× magnification.

### 2.5. Nanoemulsions Stability

An accelerated stability analysis of the nanoemulsions was conducted by centrifuging them for 30 min at 3500 rpm. The nanoemulsions’ stability was determined by monitoring the droplet size and size distribution over a 14-day period [36].

### 2.6. Preparation of Experimental Bacterial Strains

The antimicrobial activity of TTO nanoemulsions against foodborne pathogens (*Listeria monocytogenes* ATCC19112, *Escherichia coli* ATCC700728, and *Staphylococcus aureus* CMCC26003 (previously specified foodborne pathogens) were measured. Prior to testing, the bacterial culture was diluted to 10^4^ CFU/mL.

### 2.7. Determination of Minimum Inhibitory Concentration (MIC)

The MIC of TTO nanoemulsions against foodborne pathogens were determined by the two-fold dilution liquids method [35,37]. A series of two-fold dilutions of TTO nanoemulsions, ranging from 4.8% to 0.075% (*v*/*v*) were used to prepare broth media containing various concentrations of essential oils and nanoemulsions. Luria–Bertani (LB) broth with no oils and with 1% (*v*/*v*) of emulsions without TTO were used as controls. The broth medium was incubated at 37 °C for 24 h. MIC was determined as the lowest concentration of TTO nanoemulsions inhibiting the visible bacterial growth in the LB broth medium.

### 2.8. Determination of Nanoemulsions Inhibition Ability

The enhancement of bacterial-killing capacity after emulsification was measured for three model bacteria. The positive (culture medium and microorganism) and bulk controls (culture medium with bulk nanoemulsions and microorganisms) were used for the validation of the test. Blank nanoemulsions were prepared in the same way but without TTO. Bacterial growth in liquid media under different conditions was monitored in real-time by a fully automated bacterial growth detector at 37 °C. The numbers of active bacteria in samples treated with pure TTO and TTO nanoemulsions were directly monitored by LB solid medium after 8, 16, and 24 h of incubation at 37 °C [38].

### 2.9. Determination of the Antioxidant Properties of Nanoemulsions

The reaction mixtures (4.5 mL) containing 45 μL and 22.5 μL TTO nanoemulsions, 200 μmol/L FeSO_4_, 600 μmol/L salicylic acid, and 100 μmol/L H_2_O_2_ were incubated at 37 °C for 30 min (absorbance measured = 520 nm.). The control group consisted of diluted blank nanoemulsions. Calculating the ability of the compound to scavenge hydroxyl radicals was as follows:

$$Hydroxyl\;radical\;scavenging\;activity\;(\%)=[\frac{\left(A\;sample\;520\;nm-A\;control\;520\;nm\right)}{\left(A\;control\;520\;nm\right)}]\times 100$$

The reaction mixture (3 mL) containing 90 μL and 45 μL TTO nanoemulsions, and 0.1 mmol/L DPPH in ethanol solution was incubated for 30 min at 25 °C (absorbance measured = 517 nm). The control group consisted of diluted blank nanoemulsions. Calculating the ability of the compound to scavenge DPPH was as follows:

$$\;DPPH\;radical\;scavenging\;activity\;(\%)\;=\;[\frac{A\;control\;517\;nm-A\;sample\;517\;nm}{A\;control\;517\;nm}\;]\;\times \;100.$$

### 2.10. Statistical Analysis

The entire experiment was performed in triplicate, and all analyses were conducted with at least three replicates. Statistical significance was assessed by Student’s *t*-test using GraphPad Prism 8, with *p* < 0.05 considered significant.

## 3. Results and Discussion

### 3.1. Preparation and Appearance Evaluation of Nanoemulsions

According to the k value in orthogonal experiment results (Table S1), an optimal condition for TTO nanoemulsions was achieved that contained 4% surfactant, 0.5% cosurfactant (lecithin), and 75% PBS (10 mmol/L), all combined with 600 r/min for 45 min, and 420 W power ultrasound for 12 min.

**Figure 1:**
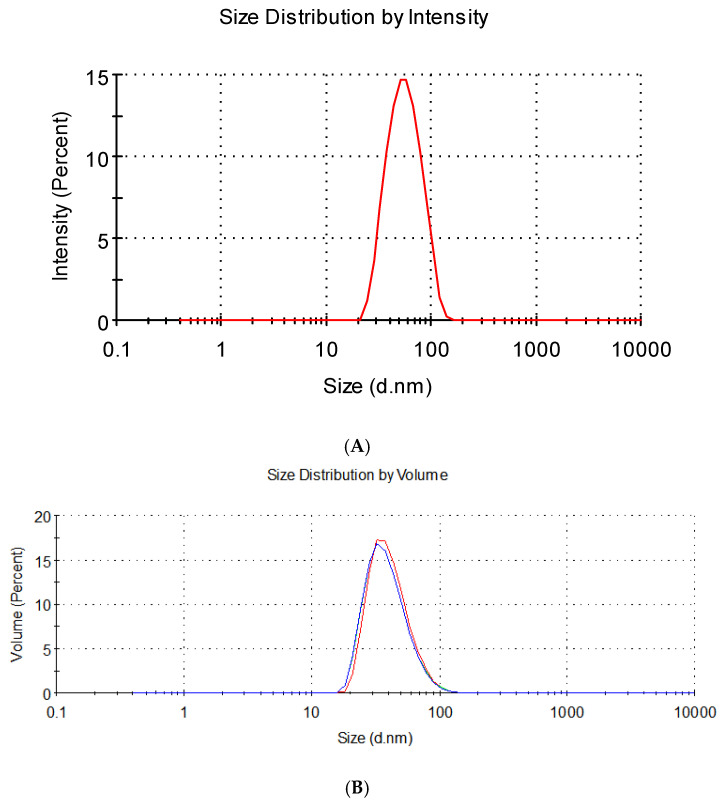
Particle size distribution of the nanoparticles. (**A**) Intensity-weighted size distribution. (**B**) Volume-weighted size distribution. The different colors correspond to different experimental groups.

The droplet size of the TTO nanoemulsions was 51.15 nm ± 0.61 nm (PDI = 0.236) (Figure 1A). Compared with some previous studies [35,39,40,41], the nanoemulsion prepared by us has a smaller particle size and lower PDI. This indicates that the system we used has great application potential, and the particle size of the refined oil-based emulsion was successfully reduced to the size of nanodroplets (51.15 nm) (Figure 1A), indicating that the functional performance of the TTO-based nanoemulsions in commercial applications had been improved.

### 3.2. TEM

**Figure 2:**
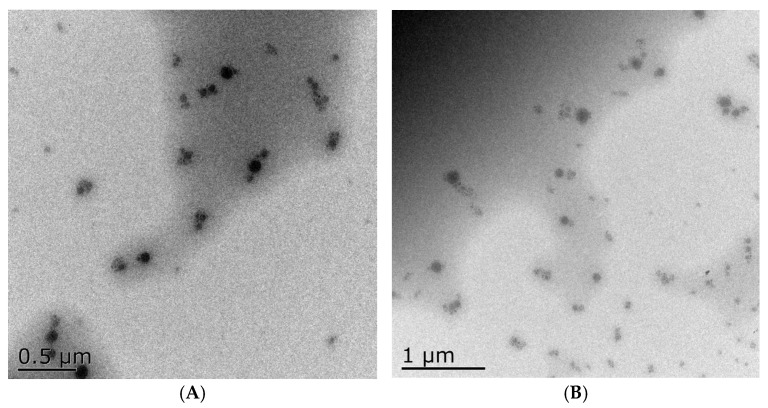
Transmission electron microscope image of nanoemulsions. The particle size distribution and aggregation of the emulsion can be observed from the images. (**A**) 20,000 times; (**B**) 10,000 times.

A transmission electron microscope magnified 30,000 times allowed us to observe the particle size distribution and morphology of the nanoemulsions. In TEM measurements of TTO nanoemulsions, droplet sizes were essentially identical to those measured by dynamic light scattering (Figure 2A,B). During measurements via light scattering, the nanoemulsion droplets tend to be encapsulated in a water film, which may result in a slight difference in measurement value.

### 3.3. Stability of the TTO Nanoemulsions

For industrial applications, nanoemulsions need to be physically stable over a long period of time. Hence, it is necessary to evaluate nanoemulsions’ stability under various conditions. We tested the stability of the TTO nanoemulsions at room temperature (Table 1). During 14 days of storage, nanoemulsions of TTO were stable to droplet growth from 51.15 to 47.79 nm (PDI 0.236 to 0.368). Compared with previous studies, the stability of our emulsion is much higher and it can be stored for a longer period of time [35,39]. There is a possibility that dynamic stability is achieved through continuous reconstruction of micelles, which could explain this slight increase in PDI. Overall, although the PDI has increased, the TTO nanoemulsions are still able to maintain a relatively uniform system (with a PDI of less than 0.5) for 14 days. The TTO nanoemulsions are still capable of being used in food preservation at ambient temperature due to their high stability [42].

### 3.4. Antimicrobial Activity of the TTO Nanoemulsions

#### 3.4.1. MIC of the TTO Nanoemulsions

There is a tendency for TTO to evaporate rapidly from surfaces as it is hydrophobic, reactive, and volatile. Therefore, emulsification is required to improve the biological activity of TTO. The results of the MIC are shown in Table 2. Compared to pure essential oils, the nanoemulsions exhibited high inhibitory effects with MICs of 0.3, 0.3, and 0.15% (*v*/*v*). In subsequent experiments, three pathogenic bacteria will be used as model bacteria to conduct inhibition experiments.

#### 3.4.2. Nanoemulsions’ Inhibition Ability

**Figure 3:**
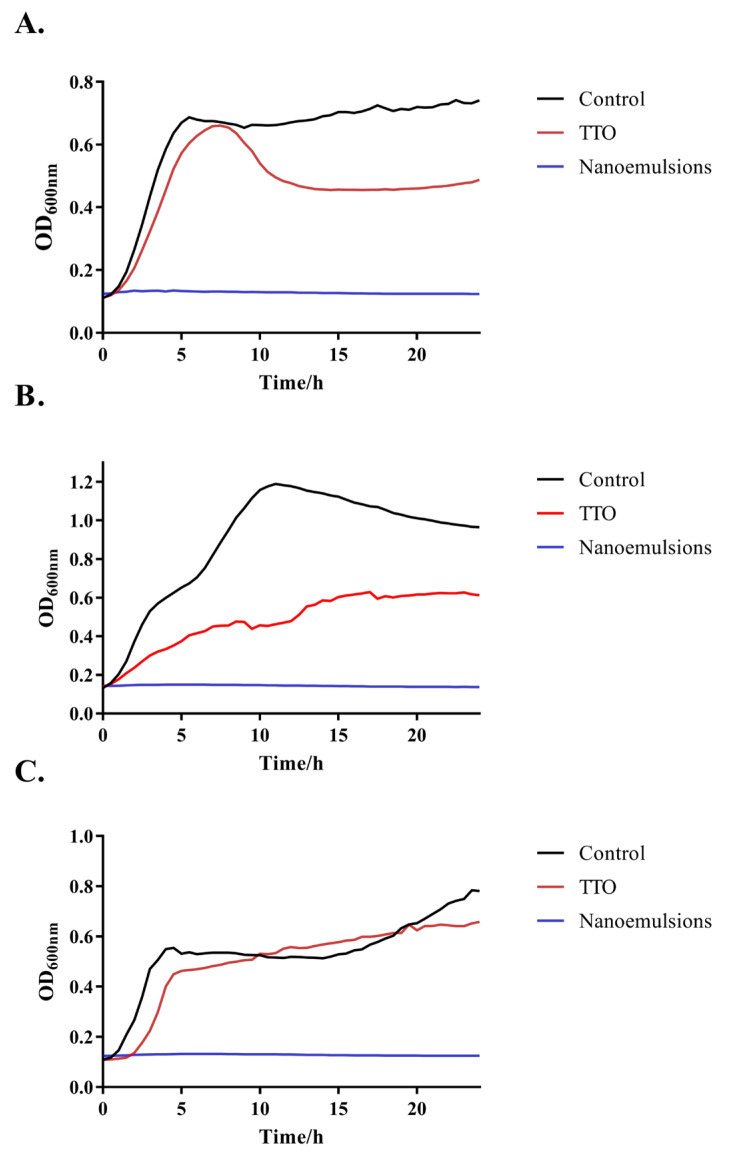
The growth curve of bacteria after adding different treatments: (**A**) *E. coli*; (**B**) *S. aureus*; (**C**) *L. monocytogenes*. Different colors represent different methods of bacterial treatment.

**Figure 4:**
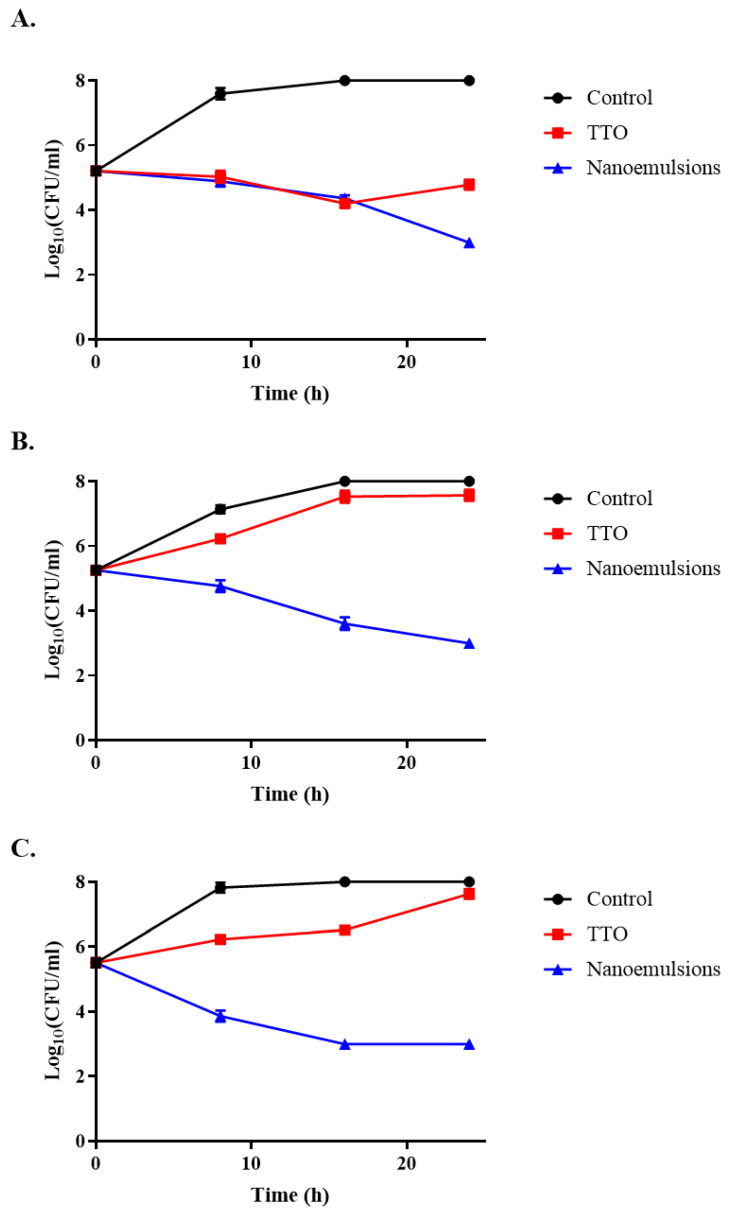
Time-kill curve: (**A**) *E. coli;* (**B**) *S. aureus*; (**C**) *L. monocytogenes.* Different shapes represent different methods of bacterial treatment.

The change in TTO’s ability to inhibit bacteria after emulsification was determined by the growth curve and colony counting. The results of the growth curve are shown in Figure 3. For *S. aureus*, pure TTO can provide some inhibition. However, it does not completely inhibit the growth of *S. aureus*. After 24 h of incubation, the OD of the medium gradually grew to over 0.5. After emulsification, the same concentration of TTO was able to completely inhibit the growth of *S. aureus*. The OD value of the medium was always maintained between 0.1 and 0.2. For *E. coli*, the TTO seems to lose its effect during the first 6 h. It was only after 6 h that TTO showed some bactericidal effects. However, when it was emulsified, TTO also showed very strong biological activity and was able to completely inhibit the multiplication of *E. coli* growth in the medium. As can be seen in Figure 3C, pure TTO does not appear to have a significant inhibitory effect on *L. monocytogenes*. *L. monocytogenes* was able to grow normally in the medium containing TTO. However, the emulsified TTO was able to completely inhibit the growth of *L. monocytogenes* at the same concentration. To obtain a more precise idea of the bacterial inhibition of TTO after emulsification, we calculated the degree of bacterial growth after 8, 16, and 24 h of incubation versus using the plate count method. The results are shown in Figure 4. We found that nano-emulsification greatly enhanced the biological activity of TTO. The nanoemulsions were able to kill bacteria rapidly and render them unable to grow within 24 h, compared with the control and pure TTO groups. The nanoemulsions exhibited potent bactericidal activity, significantly suppressing bacterial growth within 24 h compared to the control and pure TTO groups. Although TTO can inhibit bacterial growth at the beginning, it is difficult for it to play a long-term role due to its poor water solubility and strong volatilization ability. This is similar to previous studies [43,44], indicating that nano-emulsification greatly enhances the antibacterial bioactivity of TTO.

### 3.5. Antioxidant Activity of the TTO Nanoemulsions

**Figure 5:**
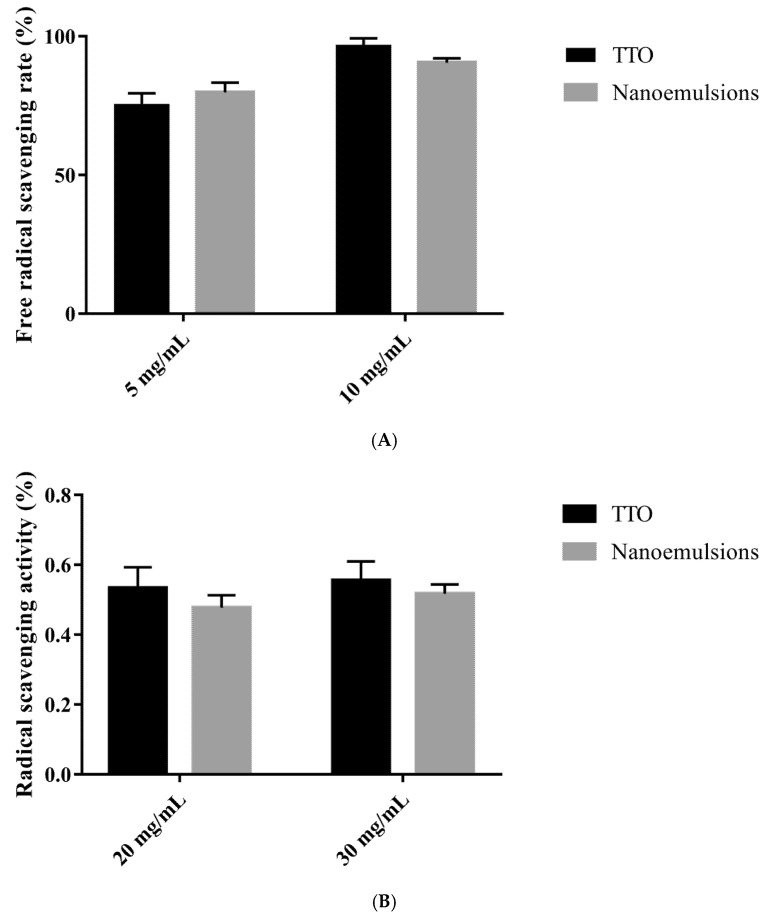
Changes in TTO antioxidation after emulsification (*p* > 0.05). (**A**) Free radical scavenging ability; (**B**) Radical scavenging ability.

In fact, in addition to the life activity of microorganisms capable of damaging the quality of food, the oxidation of oils and fats is also a problem that must be faced in the storage and preservation of foodstuffs. In production practice, antioxidants are often added to slow down the rate of oxidation in foods. Antioxidants can inhibit oxidation through free radical scavenging, free radical recombination, transition metal ion chelation, or electron transfer to form stable products. Essential oils serve as natural antioxidants and scavengers of radicals to prevent the oxidative deterioration of oxidizable foods. Recent studies have shown that TTO has a more powerful antioxidant capacity than some traditional antioxidants [45,46]. We also investigated whether TTO’s antioxidant properties were affected by emulsification. By testing the free radical scavenging rate of TTO nanoemulsions and pure TTO, we found a decrease in the antioxidant properties of the emulsions after the emulsification treatment (Figure 5). A similar phenomenon has also been found in previous studies; Liu et al. found that the free radical scavenging ability of the essential oil in the partially nano-emulsified form did not show significant changes [47]. Rinaldi et al.’s research also discovered this phenomenon. Under the treatment of nano-emulsification, the antioxidant properties of the essential oil did not show significant changes [48]. There are many reasons for this phenomenon, the main one is probably due to the formation of emulsion encapsulation which limits the rate of release of the TTO. Although emulsification mildly reduced the measured antioxidant capacity, the difference was not statistically significant (*p* > 0.05). TTO nanoemulsions still possessed good antioxidant capacity. Therefore, we believe that nano-emulsification does not affect the antioxidant properties of TTO. These results suggest that TTO nanoemulsions can be used as an easily accessible source of natural antioxidant additives in foods.

## 4. Conclusions

We successfully prepared small-particle-size nanoemulsions by using CMCS and TTO. After 14 days of storage at room temperature, the particle diameter and PDI were still good, indicating that the nanoemulsions have good stability. In addition, the bioactivity of TTO is improved by emulsification. The results of the present study showed that the nano-emulsification technique undoubtedly improved the antimicrobial activity of TTO against *L. monocytogenes*, *E. coli* and *S. aureus*. At the same time, the antioxidant activity of TTO was not significantly affected after emulsification. Therefore, TTO nanoemulsions can be used to preserve food that is susceptible to bacterial contamination, in addition to preventing food from oxidizing and deteriorating. Although there is still the issue of excessive dosage compared to antibiotics, the fact that TTO originates from natural plants undoubtedly helps it to be applied in various scenarios. We can take this research as a guideline to continue developing products such as antibacterial sprays for agricultural products and food coatings in the future, which are conducive to the application of emulsions.

## Figures and Tables

**Table 1:** Stability determination of nanoparticles.

Time (day)	Droplet Size (nm)	PDI
1	51.15 ± 2.8	0.236 ± 0.021
7	39.15 ± 3.5	0.289 ± 0.019
14	47.79 ± 6.2	0.368 ± 0.133

**Table 2:** Antimicrobial properties of the TTO and TTO nanoemulsions against *Staphylococcus aureus*, *Listeria monocytogenes*, and *Escherichia coli* (*n* = 3) ^a^.

	Essential Oil Concentrations (Nanoemulsions/Pure Essential Oils)						
	4.8%	2.4%	1.2%	0.6%	0.3%	0.15%	0.075%
*Staphylococcus aureus*	− ^b^/−	−/−	−/−	−/−	−/+	+/+	+/+
*Listeria monocytogenes*	−/−	−/−	−/−	−/−	−/+	+/+	+/+
*Escherichia coli*	−/−	−/−	−/−	−/−	−/−	−/+	+/+

^a^ The nanoemulsions were produced using the concentration of Test 2. ^b^ “−“ means no bacterial growth, “+” means bacterial growth.

## Data Availability

The original contributions presented in this study are included in the article/Supplementary Materials. Further inquiries can be directed to the corresponding authors.

## Supplementary Materials

The following supporting information can be downloaded at: https://www.mdpi.com/article/10.3390/foods14193405/s1, Table S1: Orthogonal experiment of nanoemulsions formulas (*n* = 3); Table S2: The composition of the *Tree tea* essential oil.
