# Supplementary material for: Nano-Emulsification Potentiates Tea Tree Oil Bioactivity: High-Stability Formulation for Dual Antimicrobial and Antioxidant Food Preservation
Source: Foods. 2025 Oct 1;14(19):3405. doi: 10.3390/foods14193405 (PMC12523386; doi:10.3390/foods14193405)
Supplement: Supplementary file 1 [file foods-14-03405-s001.zip › foods-3852715-supplementary.pdf]

Supplementary Material

Table S1. Orthogonal experiment of nanoemulsions formulas (n=3)[a]

Factor	Tween 80	Lecithin	Time	Particle Size (nm) (Mean ± SD)	PDI
Test 1	2%	0.5%	16 min	63.85± 0.21	0.226
Test 2	2%	1%	24 min	96.84 ± 0.36	0.237
Test 3	2%	1.5%	32 min	209.3 ± 0.72	0.436
Test 4	4%	0.5%	24 min	51.15 ± 0.16	0.236
Test 5	4%	1%	16 min	93.46 ± 0.28	0.251
Test 6	4%	1.5%	32 min	76.08 ± 0.34	0.175
Test 7	6%	0.5%	32 min	57.33 ± 0.31	0.225
Test 8	6%	1%	16 min	92.77 ± 0.21	0.272
Test 9	6%	1.5%	24 min	54.66 ± 0.15	0.125
k 1	123.3	57.4	77.6		
k 2	73.56	94.36	67.55		
k 3	68.25	113.33	120.01		
R	55.04	55.87	52.447		

[a]The experiment used organic phase (22%) aqueous phase (78%) and 940W ultrasonic intensity.

Table S2. The composition of the *Tree tea* essential oil

Ingredient	Content
(−)-Terpinen-4-ol	≈45%
1,8-Cineole	≈15%
